# Stepwise Surgical Treatment for Severe Elbow Contracture After Trans-olecranon Fracture-Dislocation: A Case Report

**DOI:** 10.7759/cureus.102681

**Published:** 2026-01-31

**Authors:** Mamiko Tahata, Taku Hatta, Gaku Matsuzawa, Mika Abe

**Affiliations:** 1 Orthopedic Surgery, Japanese Red Cross Ishinomaki Hospital, Ishinomaki, JPN; 2 Orthopedic Surgery, Joint Surgery, Sports Clinic Ishinomaki, Ishinomaki, JPN; 3 Orthopedics, Tome Citizen Hospital, Tome, JPN

**Keywords:** contracture, dislocation, elbow, fracture, trans-olecranon

## Abstract

Posttraumatic elbow contracture following trans-olecranon fracture-dislocation presents a complex challenge due to extensive soft tissue damage and joint stiffness. We report a case of a 46-year-old man who developed severe elbow contracture after the open reduction and internal fixation of a trans-olecranon fracture-dislocation, with a flexion-extension arc limited to 40° and forearm rotation restricted to 50°. A stepwise, three-phased surgical approach was employed. The first stage consisted of arthroscopic capsular release to address flexion-extension contracture. The second stage involved the open debridement of the proximal radioulnar joint and radial head replacement for rotational limitation. The third stage consisted of a repeat arthroscopic release to treat the residual restriction of flexion. Each procedure was tailored to address distinct mechanical contributors to the multifactorial stiffness, aiming to restore motion and alleviate pain through gradual functional recovery. At final follow-up, the patient achieved a flexion-extension arc of 125° and forearm rotation arc of 150°, with full return to pain-free daily activity. This case highlights the importance of staged intervention in managing complex elbow stiffness and suggests that individualized surgical sequencing can lead to favorable outcomes even in severe posttraumatic cases.

## Introduction

Posttraumatic elbow contracture is a common and challenging complication that can severely limit activities of daily living. Its reported incidence ranges from 3% to 20% [1-3], depending on the mechanism and severity of injury, particularly in cases involving fracture-dislocations or open fractures [4]. These injuries often result in extensive soft tissue damage, prolonged immobilization, and intra-articular adhesions, all of which contribute to the development of stiffness [5].

When conservative management fails to restore the functional range of motion (ROM), surgical intervention should be considered. Traditional open release techniques may require broad exposure and carry potential risks such as postoperative swelling, delayed rehabilitation, and excessive scar formation [6]. Arthroscopic release, although technically demanding, offers a less invasive alternative that enables the precise debridement of fibrotic tissue and capsular adhesions while minimizing surgical trauma [7,8].

Elbow stiffness develops through several interacting mechanisms, including capsular fibrosis, intra-articular adhesions, heterotopic ossification, and mechanical blockage caused by malunited or nonunited fractures [5,7]. These factors frequently coexist after high-energy trauma, making it difficult to determine the dominant contributor to motion loss in severe cases. As a result, clinical decision-making becomes particularly challenging because no single procedure can reliably address all mechanical constraints. A staged, mechanism-based approach may therefore be required to sequentially target each component of stiffness while minimizing surgical morbidity and allowing functional assessment between procedures.

In this report, we describe a case of severe elbow contracture following an open trans-olecranon fracture-dislocation. A staged surgical strategy, including arthroscopic procedures and radial head replacement, resulted in marked improvement in both elbow and forearm motion, as well as overall functional recovery.

## Case presentation

**Figure 1:**
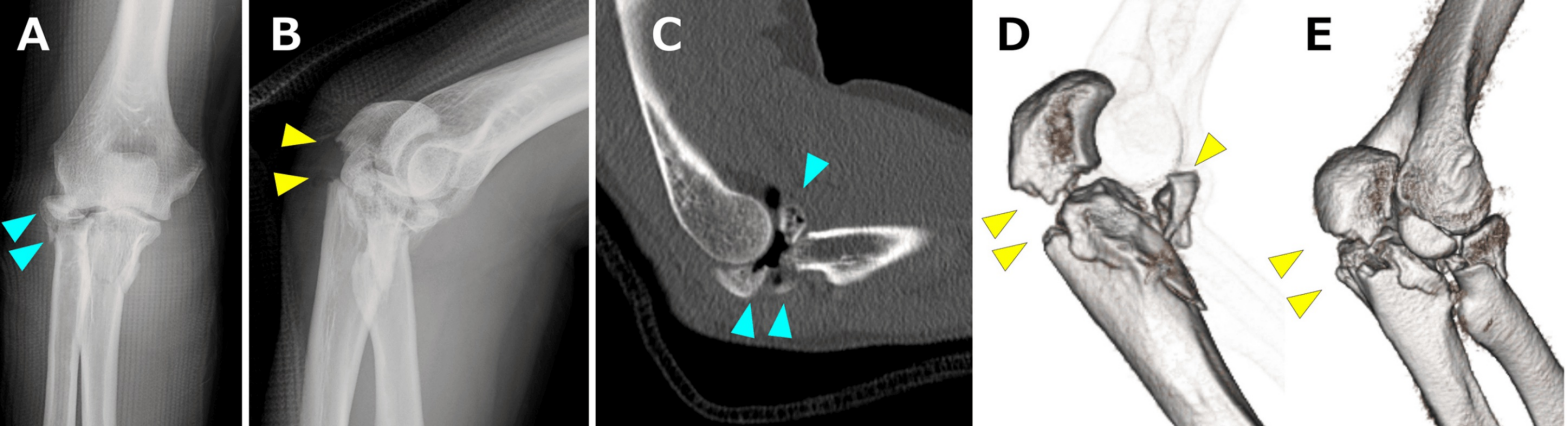
Preoperative radiographic images obtained at the referring trauma center. The plain radiographs (A, anteroposterior image; B, lateral image) and computed tomography images (C, sagittal image; D and E, three-dimensional reconstructions) were obtained at the previous trauma center, demonstrating the trans-olecranon fracture-dislocation (after reduction) with a comminuted radial head fracture (blue arrowheads) and olecranon fracture (yellow arrowheads).

**Figure 2:**
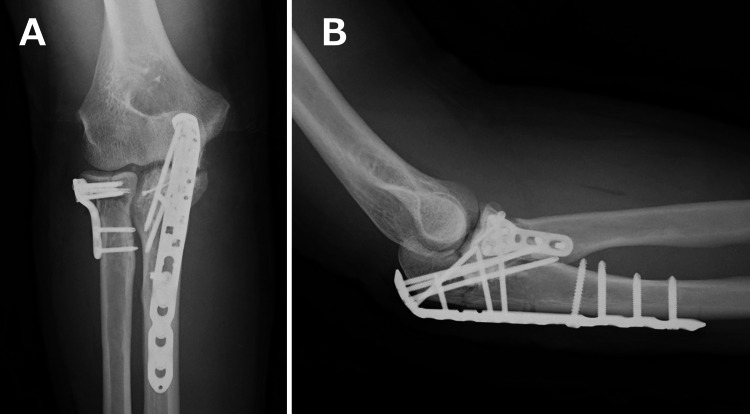
Postoperative radiographic images at a nearby trauma center. The anteroposterior image (A) and the lateral image (B) demonstrate the fixation performed at the previous trauma center, where a locking plate was applied to the radial head fracture, headless compression screws were used for the coronoid process fracture, and a locking plate was also used for the olecranon fracture. Although these findings reflected the complexity of the initial trauma, the alignment appeared acceptable at this stage.

A 46-year-old right-handed man sustained an open trans-olecranon fracture-dislocation of the left elbow after falling from a height. Initial treatment at a nearby trauma center included open reduction and internal fixation, and the preoperative radiographs and computed tomography obtained there are shown in Figure 1A-1E. A locking plate was applied to the radial head fracture, headless compression screws were used for the coronoid process fracture, and a locking plate was also used for the olecranon fracture (Figure 2A, 2B).

**Figure 3:**
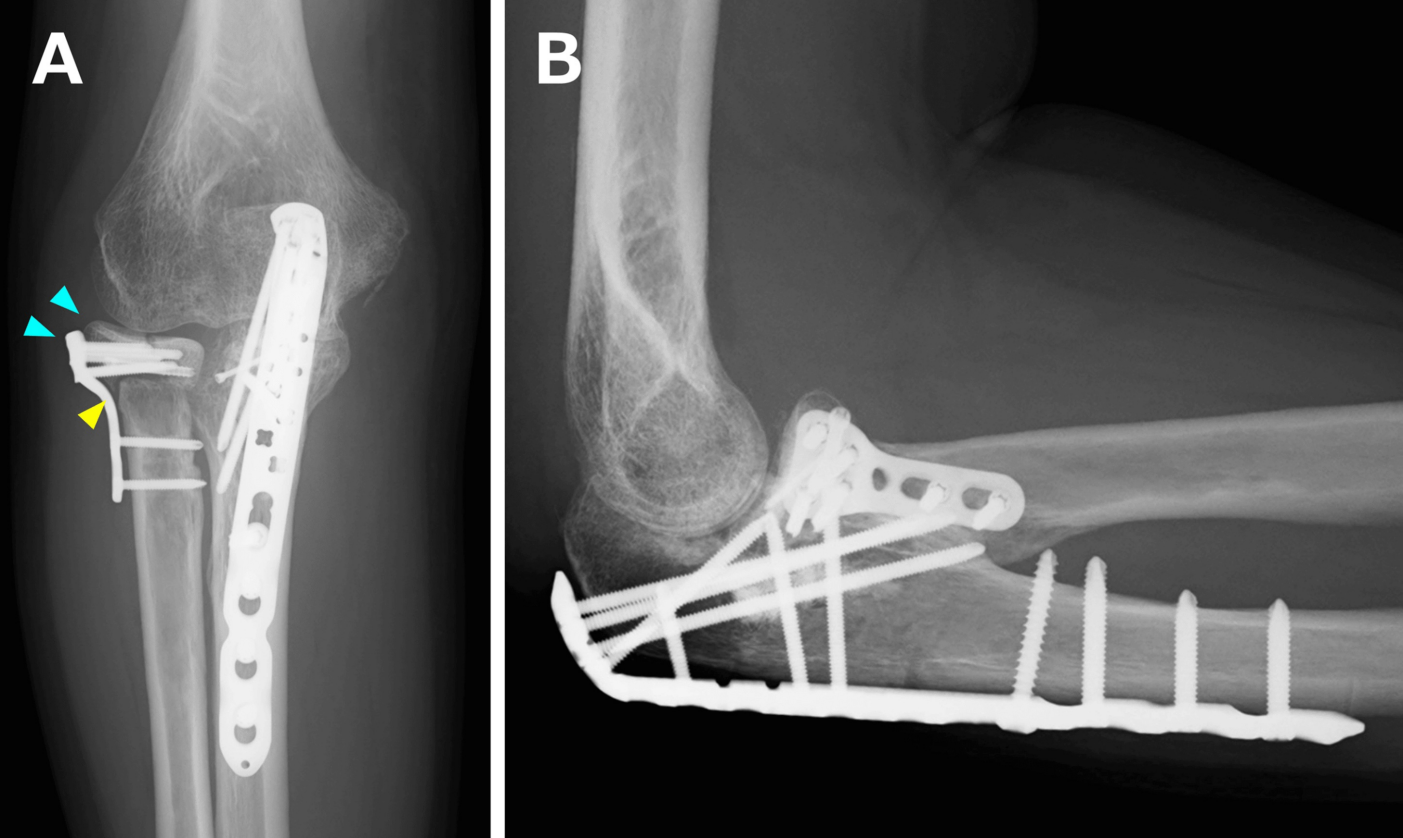
Radiographic images at initial presentation to our clinic. The anteroposterior image (A) and the lateral image (B) demonstrate the persistent nonunion of the radial head (yellow arrowhead) with associated malposition (blue arrowheads).

Despite careful rehabilitation, the patient developed progressive stiffness with severe motion pain. He was referred to our clinic five months after the initial injury. At the time of presentation, the elbow showed marked limitation in ROM, with flexion limited to 60° and extension to -20°. Forearm rotation was also restricted, with 20° of supination and 30° of pronation. The Mayo Elbow Performance Score (MEPS) was 25 points. Plain radiographs obtained at our clinic clearly demonstrated persistent nonunion of the radial head (Figure 3A, 3B). Because the diagnosis was evident on radiographs, computed tomography was not performed at our clinic. Written informed consent for the publication of this case and its accompanying images was obtained from the patient.

Treatment course

A staged surgical approach was adopted to address the severe posttraumatic contracture. Functional outcomes at each stage are summarized in Table 1.

**Table 1:** Surgical procedures and functional scores at each stage. *Pain, ROM, and MEPS represent the values at preoperative evaluation. NRS, numerical rating scale; ROM, range of motion; MEPS, Mayo Elbow Performance Score; E, extension; F, flexion; S, supination; P, pronation (degree)

Time points	Post-injury period	Pain NRS	ROM	MEPS
Stage 1	6 months	8*	E, -20; F, 60; S, 20; P, 30*	25*
Stage 2	9 months	2*	E, -15; F, 125; S, 30; P, 40*	70*
Stage 3	15 months	2*	E, -20; F, 125; S, 60; P, 70*	80*
Final follow-up	24 months	0	E, -20; F, 145; S, 70; P, 80	100

**Figure 4:**
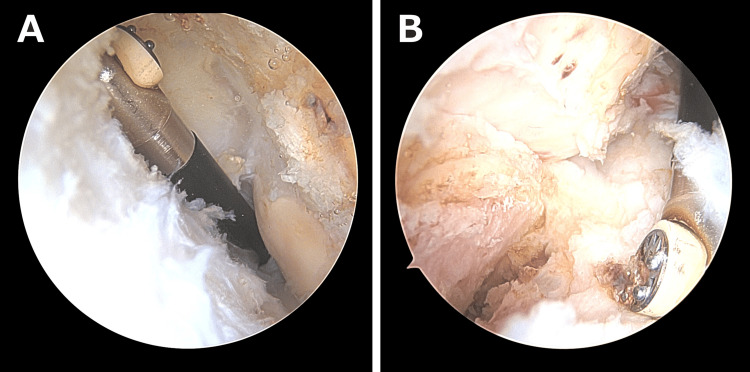
Arthroscopic images during the first procedure. The arthroscopic release of the anterior and posterior capsules was carried out to address the marked limitation in elbow motion. The images demonstrate anterior humeral capsular release (A) and capsular release around the coronoid process (B).

The first procedure was performed one month after referral to our clinic, approximately six months after the initial injury. This surgery aimed to improve elbow flexion and extension ROM. The arthroscopic release of the anterior and posterior capsules was carried out, and the anterior transposition of the ulnar nerve was additionally performed to prevent traction neuropathy during postoperative mobilization (Figure 4A, 4B). Rehabilitation was initiated in the early postoperative period, focusing on active-assisted motion exercises. The patient subsequently regained 125° of flexion and -15° of extension.

**Figure 5:**
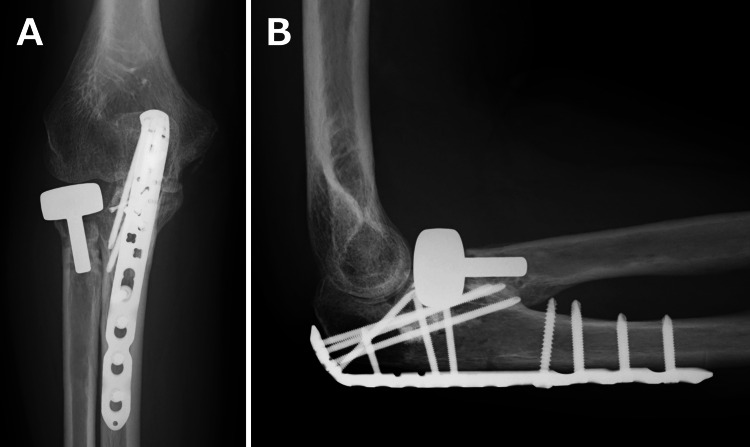
Radiographic images after the second procedure. The anteroposterior image (A) and the lateral image (B). The open debridement of the proximal radioulnar joint and the radial head replacement were performed to address persistent nonunion and mechanical impingement that limited forearm rotation. A modular radial head prosthesis (EVOLVE, Stryker Corp., Kalamazoo, MI) with a head diameter of 22 mm and a stem size of 5.5 mm was implanted.

Three months after the first procedure (nine months post-injury), a second surgery was performed to improve forearm rotation. Using a lateral (Kaplan) approach, the open debridement of the proximal radioulnar joint was performed. Because of persistent nonunion and mechanical impingement caused by malpositioned radial head fragments, radial head replacement was also performed using a modular prosthesis (EVOLVE, Stryker Corp., Kalamazoo, MI) (Figure 5A, 5B). No arthroscopic procedures were performed during this second stage. Postoperatively, supination improved to 60° and pronation to 70°.

**Figure 6:**
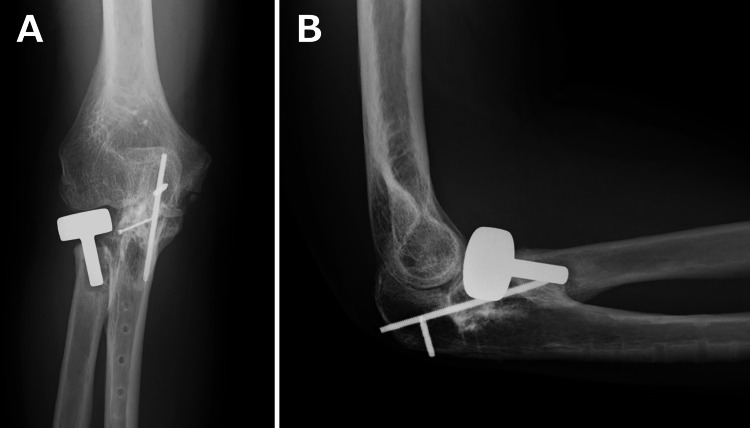
Radiographic images after the third procedure. The anteroposterior image (A) and the lateral image (B) demonstrate the removal of the olecranon locking plate, which was performed together with the repeat arthroscopic release of the anterior and posterior capsules to further improve elbow flexion.

Six months after the second procedure (15 months post-injury), a third surgery was undertaken to further enhance elbow flexion. The repeat arthroscopic release of the anterior and posterior capsules was performed, along with the removal of the olecranon locking plate (Figure 6A, 6B).

**Figure 7:**
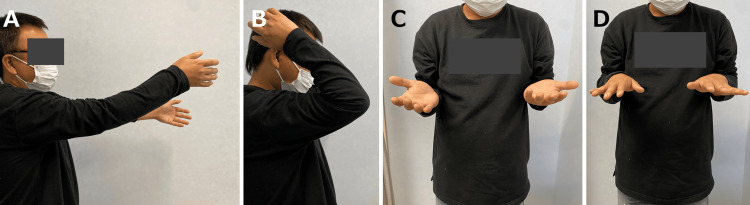
Ranges of motion at the two-year follow-up after the initial trauma. Elbow extension (A) and flexion (B). Forearm supination (C) and pronation (D).

At the final follow-up (two years after the initial trauma), the patient achieved 145° of flexion, -20° of extension, 70° of supination, and 80° of pronation (Figure 7A-7D). The Mayo Elbow Performance Score (MEPS) improved to 100 points, and the patient returned to full daily activities without pain.

## Discussion

Posttraumatic elbow contracture is a complex condition that often arises following high-energy injuries such as fracture-dislocations [4,9]. In the present case, the patient exhibited severe limitations in both flexion-extension and forearm rotation, compounded by radial head nonunion and intra-articular adhesions. To address these multifactorial constraints, we adopted a staged surgical strategy tailored to each mechanical deficit.

The first procedure focused on restoring sagittal plane motion. Arthroscopic capsular release was effective in improving flexion and extension, consistent with previous reports demonstrating the utility of arthroscopy in managing soft tissue-related stiffness [7,10]. The second procedure targeted rotational impairment, which was attributed to radial head nonunion and impingement at the proximal radioulnar joint. Careful open debridement combined with radial head replacement could lead to substantial improvement in supination and pronation. In particular, modular prostheses have been shown to yield favorable outcomes in cases of irreparable radial head fractures [8,11]. In the current case, therefore, this intervention could contribute to further treatment to treat for improving flexion and overall elbow function at the third procedure. In the final stage, repeat arthroscopic capsular release was performed to minimize residual restriction caused by capsular adhesions. This intervention resulted in functional ROM and complete pain relief.

Each stage, therefore, produced a clear and separate improvement. Flexion and extension improved after the first procedure, forearm rotation recovered after radial head replacement, and final flexion improved after the repeat arthroscopic release. These stepwise gains reflect a process of decision-making based on the specific mechanical problem identified at each stage. Imaging findings, including radial head nonunion, the incongruity of the proximal radioulnar joint, and areas of impingement, played an important role in identifying these problems and guiding the sequence of procedures. A summary of these relationships is presented in Table 2.

**Table 2:** Clinical course and key findings.

Stage	Procedure	Targeted mechanical problem	Key findings
Stage 1	Arthroscopic capsular release + open ulnar nerve transposition	Flexion-extension contracture	Dense anterior and posterior capsular fibrosis
Stage 2	Open debridement + radial head replacement	Rotational limitation due to nonunion and mechanical impingement	Nonunion fragments removed; modular prosthesis implanted
Stage 3	Repeat arthroscopic capsular release + plate removal	Residual flexion limitation	Residual capsular adhesions

This report describes a staged surgical strategy that included repeat arthroscopic release for severe posttraumatic elbow contracture. While individual techniques such as arthroscopic capsular release or radial head replacement have been reported, their integration into a phased approach tailored to distinct mechanical deficits is novel. This case highlights the potential of combining minimally invasive procedures with strategic timing to achieve functional recovery in complex elbow pathology. On the other hand, long-term follow-up will be necessary to monitor implant integrity and joint stability.

This report also has important limitations. It describes a single case without a comparison group or control subjects, and no statistical analysis can be performed. The clinical course of one patient cannot establish causality or demonstrate the superiority of this staged approach over other treatment options. The favorable outcome in this case does not indicate generalizability or guarantee a consistent safety profile.

## Conclusions

A staged surgical strategy combining arthroscopic capsular release, the open debridement of the proximal radioulnar joint, and subsequent radial head replacement proved effective in restoring functional range of motion in a patient with severe posttraumatic elbow contracture. Each procedure contributed a separate improvement, with flexion and extension improving after the first procedure, forearm rotation recovering after radial head replacement, and final flexion improving after the repeat arthroscopic release. This step-by-step recovery reflects a process of decision-making based on the specific mechanical problem identified at each stage. Imaging findings also played an important role in identifying nonunion, impingement, and capsular pathology, which helped guide the sequence of procedures. This approach may be beneficial in cases with multiple mechanical constraints, allowing for targeted correction and gradual recovery. The careful planning and timing of each intervention are essential to optimize outcomes.
